# Fibromatosis-like metaplastic carcinoma with aggressive metastasis and a TERT promoter mutation: a case report and literature review

**DOI:** 10.3389/fmed.2025.1722451

**Published:** 2025-12-18

**Authors:** Jie Chen, Yan Li, Xue Bing, Ting Wang, Wei Wang, Lei Li

**Affiliations:** 1 Jining Medical University, Jining, Shandong, China; 2 Department of Pathology, Affiliated Hospital of Jining Medical University, Jining, Shandong, China

**Keywords:** fibromatosis-like metaplastic carcinoma, breast cancer, TERT promoter mutation, metastasis, case report

## Abstract

**Background:**

Fibromatosis-like metaplastic carcinoma (FLMCa) is a rare, typically indolent subtype of triple-negative breast cancer. This report presents an exceptional case of FLMCa with an aggressive clinical course, challenging the conventional understanding of its low metastatic potential.

**Methods:**

A 57-year-old woman presented with a left breast mass. The diagnosis was confirmed via histopathology and immunohistochemistry. Sanger sequencing was used to investigate potential genetic drivers.

**Results:**

Pathological examination confirmed FLMCa, characterized by bland spindle cells and a triple-negative immunophenotype. A genetic analysis revealed a C228T mutation in the telomerase reverse transcriptase (TERT) promoter region. Despite multimodal therapy, including surgery, radiotherapy, and multiple lines of chemotherapy, the patient developed lung metastasis at 5 months and bone metastasis at 15 months post-surgery.

**Conclusion:**

This case highlights that a subset of FLMCa may exhibit unexpectedly aggressive behavior and resistance to conventional therapy. The presence of a TERT promoter mutation suggests a potential molecular mechanism for this virulence. Our findings advocate for a re-evaluation of risk stratification in FLMCa and underscore the critical need for vigilant follow-up and molecular profiling to guide future targeted therapy strategies.

## 1 Introduction

Metaplastic breast carcinoma (MBC) is a rare and aggressive group of malignancies, accounting for less than 0.5% of all breast cancers. Fibromatosis-like metaplastic carcinoma (FLMCa) is an exceptionally rare subtype of MBC, histologically characterized by a predominant proliferation of bland spindle cells that mimic benign fibrous lesions, often posing a significant diagnostic challenge (1). Historically, FLMCa has been regarded as having low potential for lymph node involvement and distant metastasis (2, 3). However, we present a case that profoundly challenges the textbook indolent narrative, documenting rapid, sequential distant metastases despite aggressive treatment in a patient with FLMCa, and we identify a TERT promoter mutation as a potential contributor to this aggressive phenotype.

## 2 Case report

We present the case of a 57-year-old woman who presented to our hospital with a 1-month history of a palpable mass in her left breast. Physical examination revealed a firm, poorly defined, and minimally mobile mass measuring approximately 7.6 cm × 5.0 cm × 5.9 cm in the upper outer quadrant. The overlying skin in the upper inner quadrant exhibited mild redness and swelling, but no ulceration, nipple discharge, or retraction was observed. No significantly enlarged axillary lymph nodes were palpable. The patient had no notable prior medical history and no family history of breast cancer.

**Figure 1:**
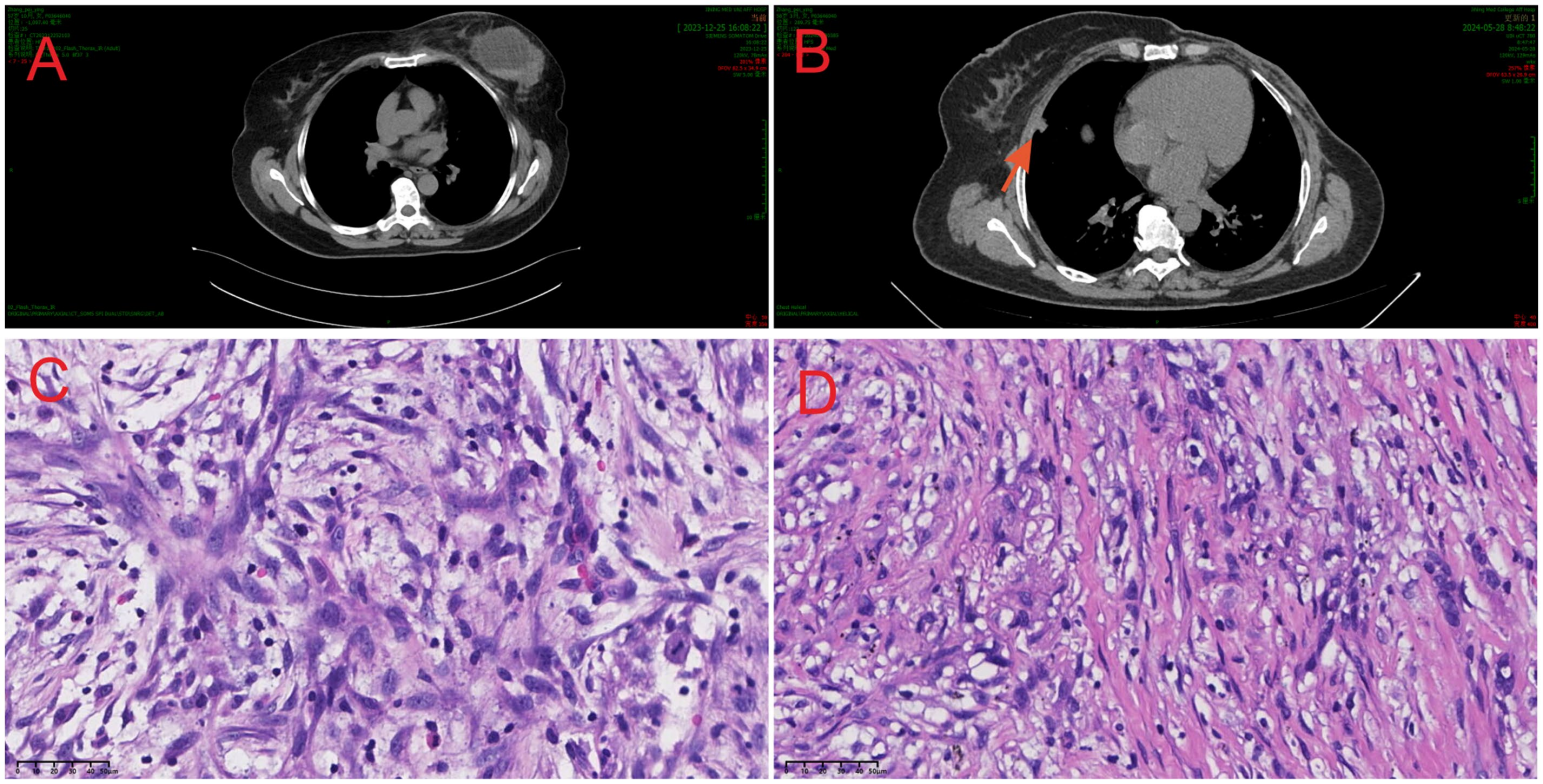
Chest CT scan, histological features of the lesions. **(A)** CT image of breast fibroma metaplastic carcinoma shows a right breast mass. **(B)** CT images show a solid nodule in the middle lobe of the right lung (red arrow). **(C)** Microscopic image of breast fibromatoid metaplastic carcinoma (HE, ×40). **(D)** Microscopic image of right lung metastatic fibromatosis-like metaplastic carcinoma (HE, ×40).

A plain chest computed tomography (CT) scan revealed a space-occupying lesion in the left breast alongside mildly enlarged left axillary lymph nodes (Figure 1A), prompting a recommendation for histopathological correlation.

Following clinical evaluation, the patient underwent a left breast segmentectomy. An intraoperative frozen section analysis described “chronic inflammatory cell infiltration and stromal spindle cell proliferation in the left breast tissue, with scattered clusters of epithelioid cells observed at the periphery,” with a definitive diagnosis deferred to permanent sections and immunohistochemistry (IHC). Consequently, a modified radical mastectomy of the left breast was performed.

**Figure 2:**
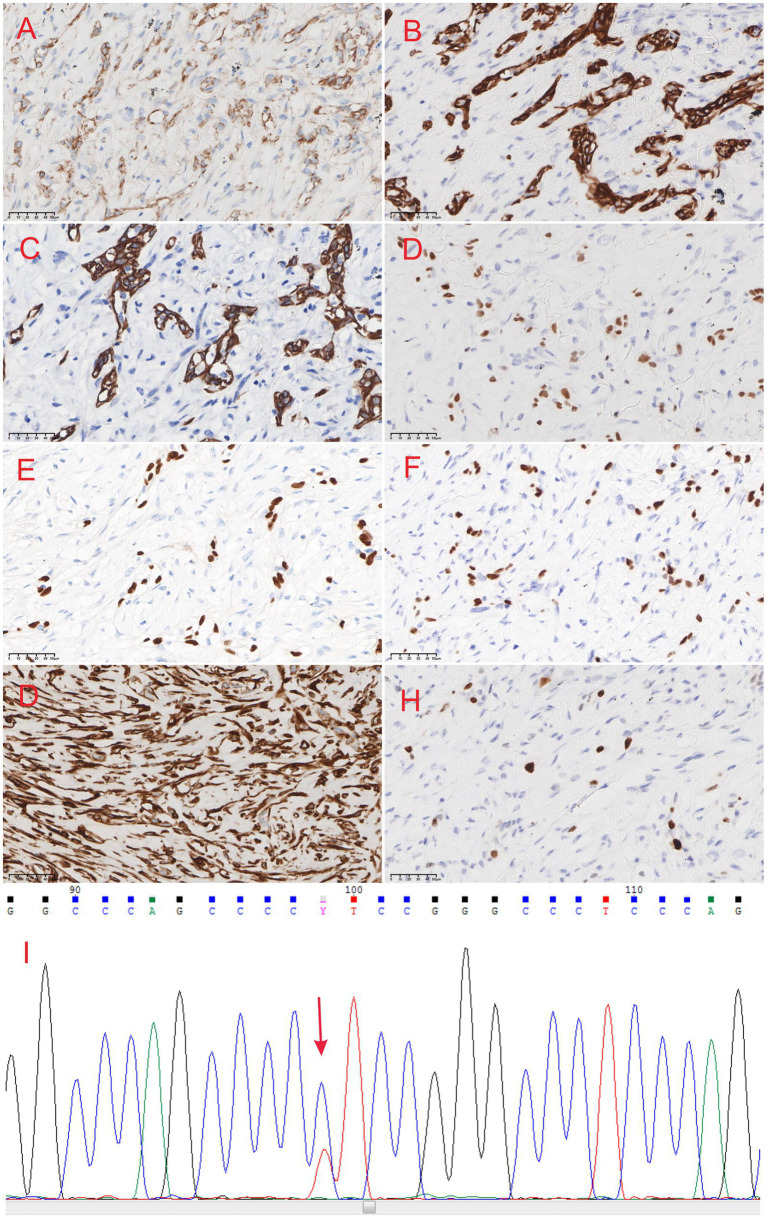
**(A)** *β*-catenin staining shows membrane-positive expression in the breast fibroma metaplastic carcinoma (×40). **(B)** CK staining shows positive expression in the cytoplasm (×40). **(C)** CK5/6 staining shows positive expression in the cytoplasm (×40). **(D)** GATA3 staining shows positive expression in the nucleus (×40). **(E)** P40 staining shows positive expression in the nucleus (×40). **(F)** P63 staining shows positive expression in the nucleus (×40). **(G)** Vimentin staining shows positive expression in the cytoplasm (×40). **(H)** KI67 staining shows positive expression in the nucleus (+,10%) (×40). **(I)**Sanger sequencing reveals a TERT C228T mutation.

Postoperative pathological examination confirmed the diagnosis of “fibromatosis-like metaplastic carcinoma.” The surrounding breast tissue exhibited localized ductal ectasia, epithelial hyperplasia, and intraductal papilloma formation, accompanied by a focal multinucleated giant cell reaction (Figure 1C). The results of immunohistochemical (IHC) staining were as follows: CK (+), vimentin (+), p40 (+), CK5/6 (+), CD34 (−), STAT-6 (−), GATA-3 (+), Ki-67 (+, 3–5%); ER (−), PR (−), beta-catenin (membranous +), and HER2 (−) (Figures 2A–H).

**Figure 3:**
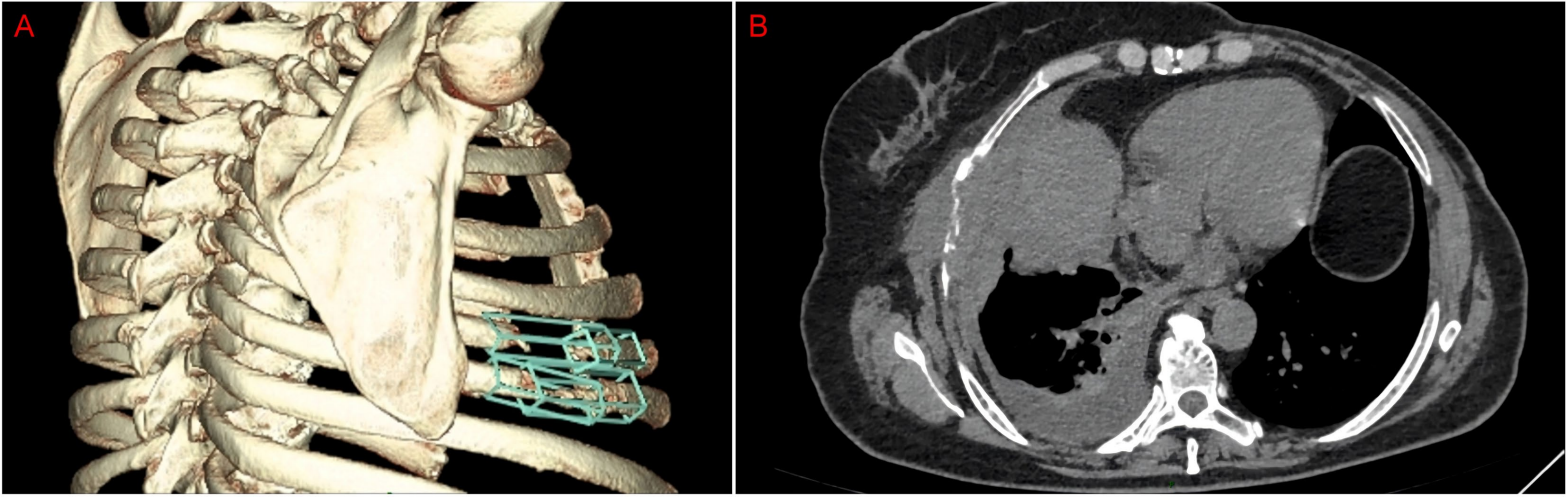
**(A)** CT images show bone destruction in the right rib. **(B)** CT shows bone destruction with a soft tissue mass.

Following mastectomy, the patient received four cycles of adjuvant chemotherapy with an EC regimen (epirubicin 80 mg on days 1–2 and cyclophosphamide 1.0 g on day 1). Twenty days after completing this regimen, a follow-up chest CT scan revealed a solid nodule in the right middle lobe, which was radiologically suspicious for a neoplastic lesion (Figure 1B). This finding was subsequently confirmed as a pulmonary metastasis by positron emission tomography—computed tomography (PET-CT) imaging. The patient underwent video-assisted thoracoscopic surgery (VATS) for partial lobectomy. An histopathological examination of the resected lung lesion confirmed the diagnosis of pulmonary metastasis from fibromatosis-like metaplastic carcinoma (FLMC) of the breast. Subsequently, she received six cycles of TX chemotherapy (docetaxel 137 mg IV on day 1 and capecitabine 2.0 g orally on days 1–14), followed by maintenance therapy with capecitabine for 3 months. Unfortunately, in March 2025, following the completion of this regimen, the patient reported pain in the right ribs. A CT scan revealed osteolytic destruction of the right fifth and sixth ribs with an associated soft tissue mass and heterogeneous density in the right fourth rib (Figure 3). Taken together, these findings were highly suggestive of metastatic disease. A biopsy of the rib lesion was performed, and the pathology was consistent with bone metastasis from the primary breast FLMC. Treatment for the bone metastases consisted of three cycles of NP chemotherapy (vinorelbine 40 mg on days 1 and 8 and cisplatin 30 mg on days 2–5). Due to disease progression, the regimen was switched to immunotherapy combined with chemotherapy (toripalimab 240 mg IV on day 0 and paclitaxel 200 mg IV on days 1 and 8) for two cycles. Subsequent re-evaluation indicated further disease progression (Figure 3).

**Figure 4:**
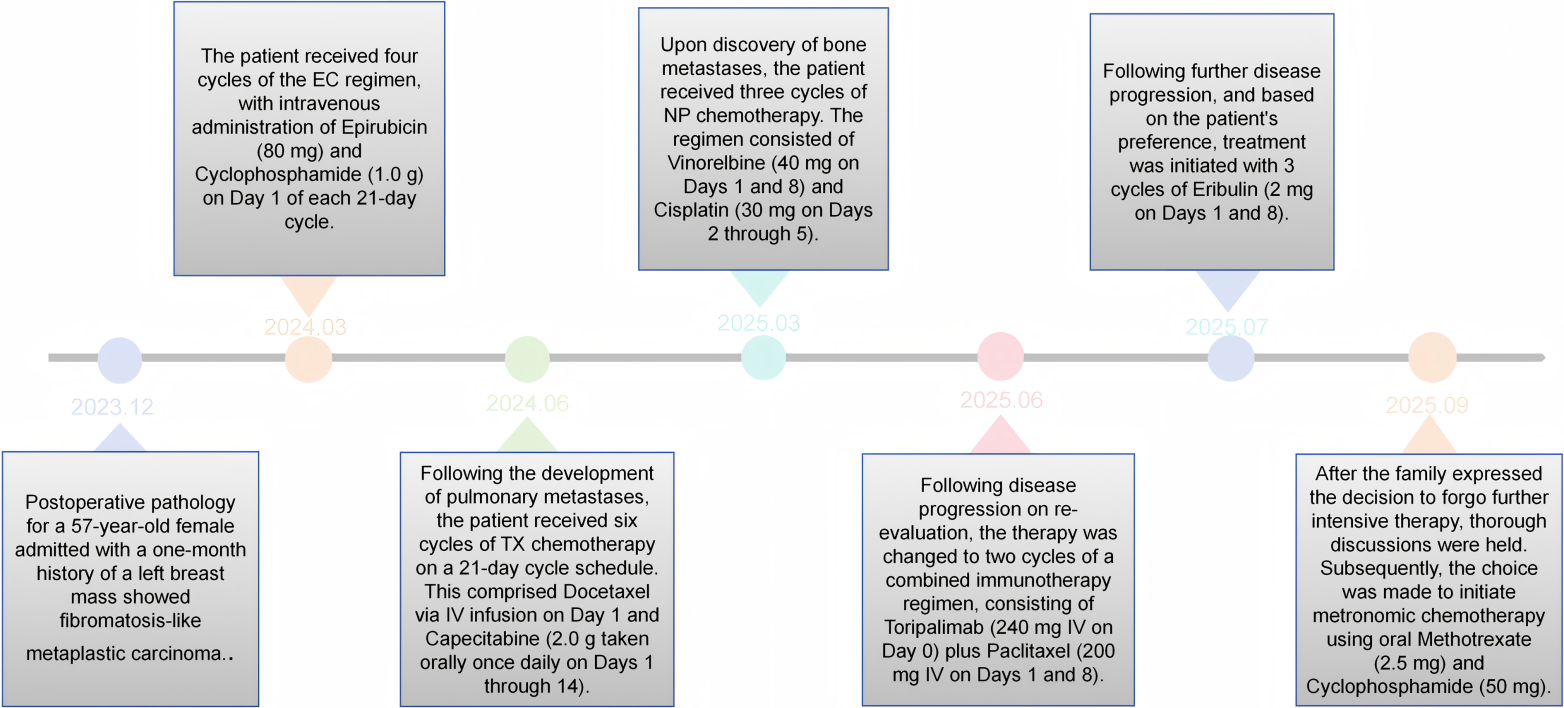
Timeline of therapeutic interventions and clinical course.

Upon the family’s request, treatment was changed to eribulin (2 mg on days 1 and 8) for three cycles; however, the disease remained uncontrolled. After detailed discussion, the family declined further aggressive treatment. A metronomic chemotherapy regimen with methotrexate and cyclophosphamide was then initiated as part of palliative care. Despite these multiple lines of treatment, the patient’s overall prognosis remained unfavorable (Figure 4).

## 3 Discussion

FLMCa is widely regarded as a locally aggressive tumor with a low propensity for lymph node involvement and distant metastasis. Although complete surgical excision with adequate margins is considered curative in the majority of cases (2, 3), recurrence often occurs after resection (4). Reports of distant metastasis remain exceedingly rare (5, 6).

The present case underscores the potential for aggressive behavior in FLMCa. Following an initial segmentectomy and subsequent completion surgery, pathological examination confirmed the diagnosis and revealed lymph node metastasis. Despite adjuvant radiotherapy and chemotherapy, the patient developed pulmonary metastasis merely 5 months post-surgery, which was histologically confirmed. The disease progressed further, with bone metastasis detected 15 months postoperatively, highlighting its metastatic potential and resistance to conventional therapies.

Histopathologically, FLMCa is a low-grade neoplasm composed of bland spindle cells with minimal mitotic activity. The tumor is predominantly composed of these spindle cells, which may be accompanied by a minor component (<5%) of glandular or squamous elements (7, 8) and, in some cases, may be mixed with conventional ductal or lobular carcinoma (9). Characteristically, the spindle cells are arranged in interlacing fascicles, storiform patterns, or wavy bundles within a variably collagenized stroma, infiltrating the surrounding breast tissue in a manner reminiscent of soft tissue fibromatosis (10).

Immunohistochemically, tumor cells consistently express p63 and CK, although CK expression may be focal in some cases. Other antibodies—ER, PgR, and HER2—are usually negative, similar to common metaplastic carcinomas. Studies by Kowalewski et al. (11), Rekhi et al. (12), Nozoe et al. (13), and others have revealed, through an immunohistochemical examination, that FLMCa does not express estrogen receptor, progesterone receptor, or HER2 receptor but primarily expresses vimentin and basal cytokeratins. In addition, an immunohistochemical expression of AE1/3 and CAM5.2 was observed, and the positive expression of epithelial and mesenchymal markers in these cells can be considered to indicate the “epithelial-mesenchymal transition” observed in these tumors. This transition leads to spindle cell metaplasia, in which neoplastic epithelial cells mimic mesenchymal stromal cells. Recognition of their epithelial nature or histogenesis depends on the demonstration of epithelial cell characteristics (14).

The majority of studies tend to diagnose FLMCa based on morphological and immunohistochemical findings (4). However, evidence suggests that TERT gene alterations play an increasingly defined role in the diagnosis of FLMCa. Although TERT promoter mutations have been described in only 0.9% of breast cancers (15), in studies by Gerald (16), Zhong (17), Krings, and Chen (18), 15 of 17 cases (88%) harbored TERT promoter alterations. We also used Sanger sequencing to detect six genes—KRAS, NRAS, HRAS, TERT, RET, and BRAF—in the tumor tissue and identified a C228T mutation in the TERT promoter region (Figure 2I). This finding indicates that TERT gene alterations may be closely related to the development of FLMCa. Additionally, genetic analysis of MED12, a gene frequently associated with phyllodes tumors of the breast, was performed, and no pathogenic mutations were detected.

Various low-grade spindle cell lesions of the breast require differential diagnosis from FLMC, including nodular fasciitis, (proliferative) scar tissue, myofibroblastoma, inflammatory myofibroblastic tumor (IMT), pseudoangiomatous stromal hyperplasia (PASH), solitary fibrous tumor, phyllodes tumor, dermatofibrosarcoma protuberans, melanoma, and primary angiosarcoma (2, 7, 19). Diagnosis requires a comprehensive approach integrating clinical presentation, imaging findings, morphological features, immunohistochemical profiling, and molecular testing. Particular emphasis should be placed on the differential diagnosis with fibromatosis, which exhibits overlapping morphological characteristics with FLMC. Fibromatosis typically exhibits proliferating spindle-shaped fibroblasts and myofibroblasts arranged in intersecting bundles, often displaying characteristic finger-like projections infiltrating surrounding breast tissue. Despite these morphological similarities, fibromatosis can be distinguished from FLMC by the absence of epithelial cell clusters and by negative immunohistochemical staining for cytokeratin (CK). In addition, another group of breast lesions with similar features to low-grade spindle cell metaplastic tumors are reactive spindle cell nodules associated with core-needle biopsy and fine-needle aspiration biopsy (20). These lesions may represent reactive processes occurring in the breast after needle aspiration biopsy. Recognizing and identifying reactive spindle cell nodule processes can help avoid overdiagnosis of malignant spindle cell tumors of the breast, especially in patients with a history of needle aspiration surgery.

FLMCa is classified as a triple-negative, basal-like breast cancer, typically demonstrating a low Ki-67 proliferation index (usually <5%) and low-grade nuclear atypia. Consequently, its prognosis is generally considered more favorable than that of high-grade triple-negative breast cancers (21). However, the occurrence of distant metastasis in our case and in the series by Lamovec et al. (5) confirms that FLMC can exhibit clinically aggressive behavior. It is therefore inappropriate to uniformly classify FLMC as low risk based merely on the statistical rarity of such cases. This warrants a re-evaluation of its management strategy toward a more vigilant and proactive approach. Triple-negative breast cancer remains the subtype with the highest rates of recurrence and mortality (22), with more than 50% of patients relapsing within 3–5 years of diagnosis (23). Although immunotherapy has advanced the treatment landscape for TNBC, its efficacy as a monotherapy remains limited. Therefore, combination strategies incorporating immunotherapy are actively being explored to improve patient outcomes (24).

This study has several limitations that should be considered. First, its design as a single-case report inherently restricts the generalizability of the findings. While it provides valuable clinical insights, larger cohort studies are necessary to confirm and extend these observations. Second, the molecular characterization of the tumor was limited in scope. Future investigations should incorporate expanded multi-omics analyses on a larger scale. Such studies would not only help validate the clinicopathological features described in this study but also contribute to the identification of novel therapeutic targets, ultimately supporting more precise and individualized treatment strategies for this rare tumor type.

## 4 Conclusion

Fibromatosis-like metaplastic carcinoma of the breast is a rare malignant tumor, conventionally considered to be of low invasiveness with a low risk of distant metastasis. However, the present case demonstrates a notably aggressive clinical course, with pulmonary and bone metastases occurring at 5 and 15 months post-surgery, respectively, despite aggressive multimodal therapy including chemotherapy and immunotherapy. This underscores that a subset of FLMCa may harbor a more aggressive biology than previously recognized.

The diagnosis of FLMCa requires a comprehensive approach, integrating characteristic histomorphology with a definitive immunohistochemical profile. Management should be multimodal, encompassing surgery, radiotherapy, chemotherapy, and, where appropriate, immunotherapy. The identified TERT promoter mutation in this case suggests that specific molecular alterations may contribute to the tumor’s invasive and metastatic potential.

Given its triple-negative phenotype and the consequent limitation of conventional targeted therapies, future research should prioritize elucidating the underlying molecular mechanisms of FLMCa. Exploring the efficacy of targeted agents and innovative immunotherapeutic strategies is essential to improve patient outcomes. Vigilant, long-term follow-up is imperative for the early detection of recurrence and metastasis. Ultimately, larger cohort studies are warranted to refine our understanding of the pathogenesis of this rare disease and to develop more effective, evidence-based treatment strategies.

## Data Availability

The datasets presented in this study can be found in online repositories. The names of the repository/repositories and accession number(s) can be found in the article/supplementary material.
